# Disparity in meat consumption: An obstacle to achieving the golden rule of the Brazilian Dietary Guidelines?

**DOI:** 10.1590/S2237-96222025v34e20240355.en

**Published:** 2025-04-11

**Authors:** Mariana Souza Lopes, Paulo Gustavo Costa e Silva Cruz, Joerika Batista Ciqueira, Cecília Furtado Craveiro, Izabele da Silva Rocha, Savio Marcelino Gomes

**Affiliations:** 1 Universidade Federal da Paraíba, Centro de Ciências da Saúde, Departamento de Nutrição, João Pessoa, PB, Brazil

**Keywords:** Observational Study, Epidemiological Surveys, Meat, Food Intake, Socioeconomic Factors, Estudio observacional, Encuestas Epidemiológicas, Carne, Consumo de Alimentos, Factores Socioeconómicos

## Abstract

**Objective:**

To investigate disparities in meat consumption in Brazil and analyze its relationship with the intake of fresh fruits and vegetables.

**Methods:**

This is a cross-sectional study using secondary data from the 2019 National Health Survey. Red meat consumption (in days) was estimated according to socioeconomic and demographic data. Odds ratios (OR) were estimated using multinomial logistic regression, and the association between meat consumption and fruit and vegetable intake was assessed by means of Poisson regression.

**Results:**

Of a total of 83,085 Brazilians participating in the study, 29.2% reported consuming meat 5-7 times per week. Sociodemographic and economic factors revealed a lower likelihood of consuming meat 5-7 days/week among women [OR 0.50, 95%CI 0.46; 0.53), older adults (OR 0.48, 95%CI 0.37; 0.63) and residents of the Northeast region of the country (OR 0.72, 95%CI 0.66; 0.79). After adjustments, red meat consumption was directly associated (p-value<0.001) with vegetable intake (0.04) and inversely associated with fruit intake (-0.04).

**Conclusion:**

Meat consumption is unequal among Brazilians and may have an impact on the consumption of fresh foods, such as fruits.

## Resumen

**Objetivo:**

Investigar las disparidades en el consumo de carne en Brasil y analizar su relación con el consumo de frutas y hortalizas frescas.

**Métodos:**

Estudio transversal que utilizó datos secundarios de la Encuesta Nacional de Salud de 2019. La frecuencia de consumo de carne roja (medida en días) se estimó con base en variables socioeconómicas y demográficas. Las razones de probabilidades (OR) se calcularon mediante regresión multinomial, y la asociación entre el consumo de carne y de frutas y hortalizas se evaluó mediante regresión de Poisson.

**Resultados:**

Del total de 83.085 brasileños participantes en el estudio, el 29,2 % informó consumir carne entre 5-7 días por semana. Al analizar los factores sociodemográficos y económicos, se observó una menor probabilidad de consumo de carne entre 5-7 días por semana en mujeres (OR 0,50; IC95% 0,46; 0,53), personas mayores (OR 0,48; IC95% 0,37; 0,63) y residentes de la región Nordeste del país (OR 0,72; IC95% 0,66; 0,79). Después de ajustes, el consumo de carne roja se asoció de forma directa con el consumo de hortalizas (0,04) e inversa con el consumo de frutas (-0,04), siendo ambas asociaciones estadísticamente significativas (p-valor<0,001).

**Conclusión:**

El consumo de carne en Brasil presenta una distribución desigual y puede influir en el consumo de alimentos frescos, como las frutas.

**Ethical aspects**

This research respected ethical principles, having obtained the following approval data:

Research ethics committee: National Research Ethics Committee

Opinion number: 3,529,376

Approval date: 8/23/2019

Certificate of submission for ethical appraisal: Not applicable

Consent form: Obtained from all participants before data collection

## Introduction

The Dietary Guidelines for the Brazilian Population recommend, as a golden rule, prioritizing natural or minimally processed foods over ultra-processed foods. And it has sustainability as one of its guiding principles ^(^ ^1^. The golden rule has been based on the associations between traditional diets - predominantly composed of natural and minimally processed foods - and healthier lifestyles ^2^ ^,^ ^3^. However, meat consumption, a key component of the Brazilian diet, may challenge golden rule in light of the principle of sustainability, as it simultaneously impacts human and environmental health or even hinders financial and economic access to other foods ^4^.

Meat consumption is a complex issue involving cultural, economic, ecological, and health factors; in addition, it has been discouraged as a strategy to mitigate the environmental impacts of food systems ^5^. Meat is a protein source of high biological value and is culturally used in various regional dishes across the country ^1^. However, its consumption may also be associated with excessive lipid intake, leading to overweight and an increased risk of chronic diseases, including cardiovascular disease and type 2 diabetes mellitus ^6^ . In turn, from an environmental perspective, meat production is linked to various negative impacts on the environment and climate ^7^. For example, in Brazil, food systems accounted for 73.7% of gross greenhouse gas emissions in 2021 ^8^. Furthermore, meat consumption practice has been questioned from an animal ethics perspective ^9^.

 The debate on reducing meat consumption is driven by Global North countries, which differ substantially Global South countries, where meat is an important resource. In Brazil, for instance, low beef consumption does not necessarily represent a socially sustainable diet, as it is associated with income inequalities, where individuals with low incomes must spend approximately 17 times more of their earnings to purchase one kilogram of meat, compared to those in the highest income group ^7^. Moreover, restricted consumption could lead to increased malnutrition in certain population groups ^10^. Social inequalities, thus further complicate achieving consensus on meat consumption recommendations for the Brazilian population that are nutritionally, culturally, ecologically, and socially appropriate. In addition, the cost of meat for socially vulnerable population groups may affect the consumption of other food groups, such as fruits and vegetables.

Socioeconomic aspects are considered in only 10% of the literature on sustainable diets ^11^, and other inequalities may arise in this context, such as racial and gender inequalities. Given this apparent impasse, the objective of this article was to investigate disparities in meat consumption in Brazil and analyze its relationship with the consumption of fresh fruits and vegetables.

## Methods

### Design

This is a cross-sectional study using data from the 2019 National Health Survey (Pesquisa Nacional de Saúde - PNS).

### Background

The PNS is a population-based survey conducted by the Instituto Brasileiro de Geografia e Estatística in partnership with the Ministry of Health. It is part of the Integrated System of Household Survey ^12^. The primary objective of the 2019 PNS was to gather information on the determinants, conditions and health needs of the Brazilian population in order to develop public policies and enhance the effectiveness of health interventions ^12^.

### Participants

The population participating in the 2019 PNS consisted of individuals aged 15 years or older, living in permanent private households. Thus, the survey sample excluded households located in special or sparsely populated census tracts, such as indigenous settlements, barracks, military installations, lodgings, camps, boats, penitentiary, penal colonies, prisons, jails, long-term care facilities for older adults, child and adolescent care networks, convents, hospitals, settlement project villages and quilombola communities ^12^.

### Variables

*Model 1: influence of socioeconomic aspects on meat consumption frequency*

The outcome variable for model 1 was red meat consumption, collected through the following question: “How many days per week do you usually eat red meat (beef, pork, lamb, goat, mutton, etc.)? The variable was categorized into consumption tertiles: 0-2 days per week, 2-4 days per week and 5-7 days per week.

The independent variables included socioeconomic characteristics: age, in years, (adolescents, adults and older adults), education level (the highest level), race/skin color (Black and non-Black), census status (urban or rural), region of the country, sex (male and female), sexual orientation (heterosexual and homo/bisexual) and income (in tertiles).

*Model 2: influence of meat consumption on the frequency of fruit and vegetable intake*

In the second model, meat consumption frequency was considered the independent variable, while the frequency of fruit and vegetable intake was considered the dependent variable. Weekly fruit and vegetable consumption was collected through the following questions: “How many days a week do you usually eat fruit?” and “How many days a week do you usually eat at least one type of vegetable?”. Thus, it was possible to analyze the association between red meat and chicken consumption and the consumption of natural foods (fruits and vegetables).

### Data source and measurement

Data collection took place between August 2019 and March 2020, using handheld computers programmed to prevent typing errors and incomplete responses. The questionnaire consisted of 26 modules, involving household information, characteristics of residents (education, work, and income), data on coverage and use of health services, health perception, accidents, lifestyle, chronic diseases, prenatal care, oral health, violence, communicable diseases, sexual activity, working conditions, and anthropometric measurements. This study used data from modules A (household), C (general characteristics of residents), D (education characteristics of residents), E (work characteristics of residents), F (household income), P (lifestyle), and Y (sexual activity).

### Bias

To ensure data quality and control, the entire data collection team underwent systematic training.

### Sample size

This study is representative of Brazil, macro-regions, urban and rural populations, and capitals. The sampling process employed a three-stage cluster random sampling method: ^1^ primary sampling units composed of one or more census tracts; ^2^ households located within the primary sampling units selected in the first stage; ^3^ an adult resident (≥15 years) selected in each household ^12^.

In order to determine the sample size and ensure the accuracy of the estimates, some indicators from the 2013 PNS were considered. These indicators included data on chronic non-communicable diseases (e.g., diabetes, hypertension and depression), violence, use of health services, health insurance coverage, smoking, physical activity and alcohol consumption, among others. The survey involved the selection of 108,525 households to participate in the interview. Among these, 90,846 interviews were conducted, resulting in a final sample. The response rate was 96.5%.

Given the complex sampling design and varying probabilities of sample selection, expansion factors (sampling weights) were defined for both selected households and residents. The final weight is the product of the inverse probabilities of selections at each sampling stage, including correction for non-responses and adjustments to population totals ^12^.

### Statistical methods

Descriptive and inferential analyses were performed. Thus, absolute and relative frequencies and their respective 95% confidence intervals (95%CI) were calculated to describe the variables.

Associations were tested using bivariate analyses by means of Pearson’s chi- square test adjusted by Rao and Scott. Finally, generalized linear models with multinomial logistic and Poisson distributions were used to test the individual and combined effect of explanatory variables on dependent variables. Odds ratio, OR) and their respective 95%CI were calculated from the coefficients estimated through the multinomial logistic models.

All analyses were performed using the R programming language, through the R Studio interface. Data were adjusted according to the respective sampling weights, using the Survey package ^13^ and PNSIBGE ^14^.

## Results

Table 1 shows the sociodemographic and economic characteristics of the population according to tertiles of red meat consumption. Approximately 25,000 (29.3%) participants reported consuming meat 5-7 times per week (third tertile of consumption). Regarding sociodemographic and economic factors, higher weekly consumption (5-7 times) was more prevalent among men compared to women (56.2% versus 43.8%, p-value<0.001); among Black individuals compared to non-Black individuals (52.4% vs. 47.6%, p-value<0.001); residents of urban areas compared to residents of rural areas (85.1% vs. 14.9%, p-value<0.001); among those with higher levels of education - higher education (20.0%), high school (37.2%) and middle school (27.7%), compared to those with elementary education or less (15.1%; p-value<0.001). As for income, individuals in the first income tertile showed less frequent meat consumption compared to those in the third tertile (16.9 vs. 50.9%, p-value<0.001). No differences were observed regarding sexual orientation.

**Table 1:** Frequency of red meat consumption in days (%) and 95% confidence interval (95%CI) according to socioeconomic factors in the population. Brazil, 2019 (n=83,085)

Variable	<2 days	2-4 days	5-7 days	p-value
**Sex**				<0.001
Male	39.3 (38.4; 40.2)	47.5 (46.6; 48.4)	56.2 (55.1; 57.2)	
Female	60.7 (59.8; 61.6)	52.5 (51.6; 53.3)	43.8 (42.8; 44.9)	
**Sexual orientation**				0.064
Heterosexual	95.3 (94.9; 95.8)	95.7 (95.2; 96.1)	94.9 (94.43; 95.48)	
Homo and bisexual	1.7 (1.4; 1.9)	1.7 (1.4; 1.9)	1.6 (1.4; 1.9)	
Other	3.0 (2.7; 3.3)	2.7 (2.34; 3.00)	3.4 (2.9; 3.9)	
**Race/skin color**				<0.001
Black	57.4 (56.3; 58.5)	52.8 (51.7; 53.9)	52.4 (51.3; 53.5)	
Non-black	42.6 (41.5; 43.7)	47.2 (46.1; 48.3)	47.6 (46.5; 48.7)	
**Education level**				<0.001
Elementary or lower education	18.2 (17.4; 19.1)	14.7 (14.0; 15.4)	15.1 (14.3; 15.9)	
Middle school	29.0 (28.0; 29.9)	25.6 (24.6; 26.6)	27.7 (26.6; 28.7)	
High school	35.2 (34.2; 36.2)	36.9 (35.9; 37.9)	37.2 (36.0; 38.3)	
Higher education	17.6 (16.6; 18.6)	22.8 (21.6; 23.9)	20.0 (18.8; 21.2)	
**Area**				<0.001
Urban	86.2 (85.6; 86.7)	87.2 (86.5; 87.7)	85.1 (84.4; 85.9)	
Rural	13.8 (13.2; 14.5)	12.9 (12.3; 13.5)	14.9 (14.2; 15.6)	
**Region**				<0.001
North	8.5 (8.1; 8.9)	7.0 (6.7; 7.4)	6.6 (6.2; 6.9)	
Northeast	30.9 (29.9; 31.7)	25.2 (24.3; 26.1)	19.9 (18.9; 20.7)	
Southeast	44.3 (43.2; 45.5)	44.5 (43.3; 45.7)	41.1 (39.9; 42.4)	
South	12.2 (11.6; 12.8)	16.4 (15.7; 17.1)	19.3 (18.4; 20.2)	
Midwest	4.2 (3.9; 4.4)	6.9 (6.5; 7.3)	13.2 (12.5; 13.9)	
**Income**				<0.001
1st tertile	26.1 (25.1; 26.9)	19.8 (19.0; 20.5)	16.9 (16.6; 17.6)	
2nd tertile	32.9 (31.9; 33.8)	31.4 (30.4; 32.4)	32.9 (31.2; 33.2)	
3rd tertile	41.1 (40.0; 42.2)	48.8 (47.6; 50.1)	50.9 (49.7; 52.7)	
**Age**				<0.001
Adolescents	3.8 (3.4; 4.2)	4.2 (3.8; 4.6)	4.6 (4.2; 5.1)	
Adults	69.4 (68.5; 70.4)	73.6 (72.6; 74.5)	73.6 (72.6; 74.5)	
Older adults	26.8 (25.8; 27.7)	22.2 (21.3; 23.1)	21.8 (20.9; 22.7)	

Table 2 shows that the groups with the lowest likelihood of consuming meat 5-7 days/week were women [odds ratio (OR) 0.50, p-value<0.001), older adults (OR 0.48, p-value<0.001) and residents of the Northeast region of the country (OR 0.72, p-value<0.001). On the other hand, the highest likelihood of consuming meat 5-7 days/week was observed among individuals from the South (OR 1.63, p-value<0.001) and Midwest (OR 3.62, p-value <0.001) regions, those with higher income (OR 1.65, p-value<0.001), those with complete high school (OR 1.14, p-value<0.014) and who lived in rural areas (OR 1.37, p-value<0.001) (Table 2).

**Table 2:** Odds ratio (OR) and 95% confidence interval (95%CI) for meat consumption according to sociodemographic variables in the population. Brazil, 2019 (n=83,085)

Variable	Frequency of red meat consumption			
	2-4 days	p-value	5-7 days	p-value
	OR (95%CI)		OR (95%CI)	
**Sex**				
Male	1.00		1.00	
Female	0.70 (0.66; 0.74)	<0.001	0.50 (0.46; 0.53)	<0.001
**Age (years)**				
Adolescents (0-19)	1.00		1.00	
Adults (19-60)	0.79 (0.61; 1.02)	0.074	0.66 (0.52; 0.85)	0.001
Elderly (≥60)	0.63 (0.49; 0.82)	<0.001	0.48 (0.37; 0.63)	<0.001
**Race/skin color**				
Non-Black	1.00		1.00	
Black	0.95 (0.89; 1.01)	<0.11	0.95 (0.89; 1.02)	<0.200
**Education level**				
Elementary education or less	1.00		1.00	
Middle school	1.03 (0.93; 1.13)	0.6	1.08 (0.97; 1.19)	0.2
High school	1.18 (1.07; 1.30)	<0.001	1.14 (1.03; 1.27)	<0.014
Higher education	1.36 (1.21; 1.52)	<0.001	1.11 (0.98; 1.25)	0.110
**Area**				
Urban	1.00		1.00	
Rural	1.10 (1.0; 1.19)	0.016	1.37 (1.26; 1.50)	<0.001
**Region**				
Southeast	1.00		1.00	
North	0.87 (0.79; 0.95)	0.003	0.86 (0.77; 0.96)	0.007
Northeast	0.88 (0.82; 0.96)	0.002	0.72 (0.66; 0.79)	<0.001
South	1.33 (1.22; 1.46)	<0.001	1.63 (1.48; 1.80)	<0.001
Midwest	1.67 (1.51; 1.86)	<0.001	3.62 (3.23; 4.04)	<0.001
**Income (tertile)**				
1st	1.00		1.00	
2nd	1.20 (1.10; 1.30)	<0.001	1.40 (1.29; 1.52)	<0.001
3rd	1.32 (1.20; 1.44)	<0.001	1.65 (1.50; 1.82)	<0.001

After adjusting for sex, age, education, census area, region of the country and income, meat consumption was inversely associated with fruit consumption (β -0.04) and directly associated with vegetable consumption (β 0.04) (Table 3).

**Table 3:** Estimate (β) and standard deviation (SD) of the frequency of fruit and vegetable consumption according to the frequency of red meat consumption in the population. Brazil, 2019 (n=83,085)

	Fruits	Vegetables		
	β (SD)	β adjusted^a^ (SD)	β (SD)	β adjusted^a^ (SD)
Frequency of red meat consumption (days/week				
0-1	1	1	1	1
2-4	0.01 (0.01)	0.01 (0.01)	0.05 (0.01)^b^	0.04 (0.01)^b^
5-7	-0.05 (0.01)^b^	-0.04 (0.01)^b^	0.05 (0.01)^b^	0.04 (0.01)^b^

Notes: ^a^Adjusted for sex, age, education level, census area, region of the country and income; bp -value<0.001

## Discussion

Data analysis revealed disparities in red meat consumption among Brazilians based on sex, race/skin color, region and income, and that red meat consumption may have negatively impacted fruit intake. Women, older adults and people living in the North and Northeast regions of the country were less likely to consume red meat. In contrast, individuals with higher incomes who lived in the Midwest region of the country were more likely to consume red meat compared to their counterparts. 

Disparities in meat consumption show that the greatest environmental impact related to meat consumption is concentrated primarily among social groups with higher levels of privilege. The findings of this article reinforce the relationship between sociocultural aspects and meat consumption, showing greater consumption among White men who have high-income living in economically developed regions. Although the environmental impact of meat consumption was not directly evaluated in this study, meat is among the foods that most contribute to the negative environmental impacts of the food system, with high consumption being directly related to poorer environmental indicators ^6^.

The positive association between red meat and vegetable consumption indicated that the higher the red meat consumption, the higher the vegetable consumption. This finding supports the hypothesis that in Brazil, red meat is culturally consumed during main meals, such as lunch and dinner, and is associated with culinary preparations ^15^. Conversely, the negative association between red meat and fruit intake can be explained by the high cost of meat for lower-income groups ^7^. Thus, fruit may be overlooked in favor of purchasing meat or other foods that provide higher energy intake and greater social value on the Brazilian plate ^15^ ^,^ ^16^.

Recommendations to reduce meat consumption, however, cannot be universal, given the profound social inequalities in Brazil. Diets with lower frequency of red meat consumption among women and people living in the North and Northeast regions may be related to the impact of social inequalities on food and nutrition in Brazil. Food insecurity and reduced meat consumption are correlated ^17^. In 2021/2022, 58.7% of Brazilian households experienced food insecurity. Of these, 19.3% were female-headed households who experienced food insecurity. This figure was nearly higher 8% than that observed in male-headed households. Furthermore, the highest prevalence of food insecurity was measured in the North (45.2%) and Northeast (38.4%) regions ^18^. Therefore, recommendations on meat consumption should consider two distinct messages for social groups at opposite poles: 1) ensuring food and nutritional security for vulnerable populations who rely on meat for essential nutrients; and 2) promoting the reduction of meat consumption for groups experiencing food and nutrition security, who have means to make sustainable dietary choices.

Income-related disparities were also observed. Individuals in the third income tertile were more likely to consume red meat 5-7 times per week compared to those in the first tertile. This trend is observed across Latin America, where income growth drives meat consumption more rapidly than in wealthier countries ^19^. Although new evidence suggests that, globally, the relationship between meat consumption and income follows an inverted U-shaped, the inflection point is so high that it is above the 90th percentile for per capita income ^20^. In practical terms, interventions to reduce meat consumption should focus on populations with higher incomes.

On the other hand, the relationship between age and a lower likelihood of meat consumption does not seem to be justified by social injustice. Research indicates that older adults tend to eat less meat due to various factors. Sensory perception and food preferences change with age, affecting meat consumption. Difficulty in eating meat products, especially those with tougher textures, is a common issue for older adults ^21^.

Developing national dietary recommendations based on sustainability is a challenge in contexts of social inequality. Meat simultaneously challenges two principles of the Dietary Guidelines for the Brazilian Population: the consumption of natural foods and food sustainability. Red meat consumption is directly linked to higher risks of excessive saturated fat and sodium intake, but helps prevent deficiencies in essential nutrients, such as proteins, iron, zinc and vitamin B12 ^22^. On the other hand, to meet the principle of environmental sustainability, meat consumption must be limited, as it accounts for most greenhouse gas emissions and water use in food systems ^5^. However, restricting meat consumption could increase malnutrition by 10% in vulnerable groups ^10^. Communicating this paradigm to the Brazilian population is among the urgent challenges for promoting healthy and sustainable eating.

However, these findings should be analyzed considering some limitations. The primary limitation of this study is the information bias inherent in self-reported consumption frequency, a characteristic of the PNS. This does not limit the strength and interpretation of the results of the article, which represent one of the first studies, using a national sample, on inequalities in red meat consumption and associated factors.

Despite the methodological limitations, the study highlights the importance of using large national databases, such as the PNS, to conduct comprehensive and representative analyses. The use of a robust national sample and the application of advanced statistical methods strengthen the validity of the results and their relevance for health policy formulation.

In conclusion, this study provides valuable insights into the relationship between sociodemographic factors and dietary patterns, and suggests future avenues for analysis. Its results enable broader public health implications for the country by assessing demographic and socioeconomic factors, while highlighting challenges to adhering to the Dietary Guidelines’ golden rule and principles amid social and economic inequalities.

Therefore, this information is essential, alongside data on meat expenditure according to inequalities, for the development of targeted public policies and health interventions aimed at reducing disparities and promoting healthier eating habits.

Furthermore, the relationship identified between red meat consumption and the consumption of natural foods, such as fruits and vegetables, provides a basis for promoting an adequate, healthy and sustainable diet. By understanding food consumption patterns and their influencing factors, policymakers can develop strategies to encourage the substitution of red meat with healthier and more sustainable options, thereby improving public health and environmental sustainability. Thus, this perspective is especially relevant in the current context, in which chronic non-communicable diseases and environmental degradation are growing concerns.

## Data Availability

Not applicable.
