# Supplementary material for: Disparity in meat consumption: An obstacle to achieving the golden rule of the Brazilian Dietary Guidelines?
Source: Epidemiol Serv Saude. 2025 Apr 11;34:e20240355. doi: 10.1590/S2237-96222025v34e20240355.en (PMC11998654; doi:10.1590/S2237-96222025v34e20240355.en)
Supplement: Translation

# Translation: Disparidade no consumo de carne: um obstáculo para alcance da regra de ouro do Guia Alimentar para a População Brasileira?

**Authors:** Mariana Souza Lopes, Paulo Gustavo Costa e Silva Cruz, Joerika Batista Ciqueira, Cecília Furtado Craveiro, Izabele da Silva Rocha, Savio Marcelino Gomes

**Affiliations:** 1 Universidade Federal da Paraíba, Centro de Ciências da Saúde, Departamento de Nutrição, João Pessoa, PB, Brazil

**Keywords:** Estudo observacional, Inquéritos Epidemiológicos, Carne, Ingestão de Alimentos, Fatores Socioeconômicos

## Abstract

**Objetivo:**

Investigar as disparidades no consumo de carnes no Brasil e analisar sua relação com o consumo de frutas e hortaliças in natura.

**Métodos:**

Estudo transversal com dados secundários da Pesquisa Nacional de Saúde 2019. O consumo de carne vermelha (em dias) foi estimado segundo dados socioeconômicos e demográficos. Razões de chances (OR) foram estimadas por regressão multinomial, e a associação entre consumo de carne e de frutas e hortaliças, por regressão de Poisson.

**Resultados:**

Do total de 83.085 brasileiros participantes do estudo, 29,2% relataram consumir carne 5-7 vezes na semana. Quando investigados os fatores sociodemográficos e econômicos, foi observada menor chance de consumo de carne igual a 5-7 dias/semana entre o de mulheres [OR 0,50, IC95% 0,46; 0,53), idosos (OR 0,48, IC95% 0,37; 0,63) e os residentes do Nordeste do país (OR 0,72, IC95% 0,66; 0,79). Após ajustes, o consumo de carne vermelha foi associado (p-valor<0,001) de forma direta ao consumo de hortaliças (0,04) e inversa ao de frutas (-0,04).

**Conclusão:**

O consumo de carne é desigual entre os brasileiros e pode repercutir sobre o consumo de alimentos in natura, como as frutas.

**Aspectos éticos**

A presente pesquisa respeitou os princípios éticos, obtendo os seguintes dados de aprovação:

Comitê de ética em pesquisa: Comissão Nacional de Ética em Pesquisa

Número do parecer: 3.529.376

Perspectivas: 3.529.376

Certificado de apresentação de apreciação ética: Não se aplica

Termo de consentimento: Obtido de todos os participantes antes da coleta

## Introdução

O Guia Alimentar para a População Brasileira preconiza, como regra de ouro, a preferência por alimentos in natura ou minimamente processados em relação a alimentos ultraprocessados. E possui a sustentabilidade como um dos princípios norteadores ^1^. A regra de ouro tem sido fundamentada nas associações entre dietas tradicionais - compostas majoritariamente por alimentos in natura e minimamente processados - e modos de vida mais saudáveis ^2^ ^,^ ^3^. No entanto, o consumo de carnes, um importante item na dieta brasileira, pode colocar a regra de ouro em confronto com o princípio da sustentabilidade, por impactar simultaneamente a saúde humana e ambiental ou mesmo desafiar o acesso financeiro e econômico a outros alimentos ^4^. 

O consumo de carnes é complexo e envolve fatores culturais, econômicos, ecológicos e de saúde; além disso tem sido desestimulado como estratégia para mitigar os impactos ambientais dos sistemas alimentares ^5^. As carnes são uma fonte proteica de alto valor biológico e culturalmente utilizadas em diversas preparações regionais pelo país ^1^. No entanto, o seu consumo também pode se associar à ingestão excessiva de lipídios, com efeitos sobre excesso de peso e aumento do risco de doenças crônicas, incluindo doenças cardiovasculares e diabetes tipo 2 ^6^. Por sua vez, na perspectiva ambiental, a produção de carne se relaciona a diversos impactos negativos no meio ambiente e no clima ^7^. Por exemplo, no Brasil, os sistemas alimentares foram responsáveis por 73,7% das emissões brutas de gases de efeito estufa em 2021 ^8^. Ademais, a prática de consumir carnes tem sido questionada do ponto de vista da ética animal ^9^.

O debate sobre a redução do consumo de carnes é liderado por países do Norte Global, que guardam profundas diferenças em relação a países do Sul Global, onde a carne é um recurso importante. No Brasil, por exemplo, o consumo baixo de carne bovina não representa necessariamente uma dieta sustentável do ponto de vista social, pois está associado a desigualdades de renda, em que indivíduos com baixos rendimentos precisam gastar cerca de 17 vezes mais de sua renda para comprar um quilo de carne, em comparação com pessoas do grupo de renda mais alta ^7^. Ainda, a restrição de consumo pode estar associada ao aumento da desnutrição em alguns grupos populacionais ^10^. As desigualdades sociais, portanto, tornam ainda mais complexo um consenso sobre a recomendação do consumo de carnes para a população brasileira, de forma que seja adequada do ponto de vista nutricional, cultural, ecológico e social. Além disso, o custo da carne para grupos populacionais em vulnerabilidade social pode repercutir sobre o consumo de outros grupos alimentares, como as frutas e hortaliças.

Os aspectos socioeconômicos são considerados em apenas 10% da literatura sobre dietas sustentáveis ^11^, e outras desigualdades podem emergir neste contexto, a exemplo das desigualdades raciais e de gênero. Diante desse aparente impasse, o objetivo desse artigo foi investigar as disparidades no consumo de carnes no Brasil e analisar sua relação com o consumo de frutas e hortaliças in natura.

## Métodos

### Delineamento do estudo 

Trata-se de estudo transversal, com dados da Pesquisa Nacional de Saúde (PNS) de 2019. 

### Contexto

A PNS é um inquérito de base populacional conduzido pelo Instituto Brasileiro de Geografia e Estatística em parceria com o Ministério da Saúde, e faz parte do Sistema Integrado de Pesquisas Domiciliares ^12^. O objetivo geral da PNS 2019 foi levantar informações sobre os determinantes, condicionantes e necessidades de saúde da população brasileira, com vistas à elaboração de políticas públicas e maior efetividade nas intervenções em saúde ^12^.

### Participantes

A população participante da PNS 2019 consistiu de indivíduos com idade maior ou igual a 15 anos de idade, residentes em domicílios particulares permanentes. Logo, a amostra da pesquisa excluiu os domicílios localizados em setores censitários especiais ou de escassa população, como agrupamentos indígenas, quartéis, bases militares, alojamentos, acampamentos, embarcações, penitenciárias, colônias penais, presídios, cadeias, instituições de longa permanência para idosos, redes de atendimento integrado à criança e ao adolescente, conventos, hospitais, agrovilas de projetos de assentamento e agrupamentos quilombolas ^12^.

### Variáveis

*Modelo 1: influência de aspectos socioeconômicos na frequência do consumo de carnes*

A variável desfecho do modelo 1 foi o consumo de carnes vermelhas, coletado através da seguinte pergunta: “Em quantos dias da semana o(a) Sr(a) costuma comer carne vermelha (boi, porco, cabrito, bode, ovelha etc.)?”. A variável foi dividida em tercis de consumo: 0-2 dias por semana, 2-4 dias por semanas e 5-7 dias por semana.

Como variáveis independentes, foram consideradas as variáveis socioeconômicas de idade em anos (adolescentes, adultos e idosos), escolaridade (grau mais elevado), raça/cor da pele (negros e não negros), situação censitária (urbano ou rural), região do país, sexo (masculino e feminino), orientação sexual (heterossexual e homo/bissexual) e renda (em tercis). 

*Modelo 2: influência do consumo de carnes na frequência do consumo de frutas e hortaliças*

No segundo modelo, a frequência do consumo de carnes foi considerada como variável independente, e a frequência do consumo de frutas e verduras e legumes, como dependente. O consumo de frutas e legumes semanal foi coletado através das perguntas seguintes: “Em quantos dias da semana o(a) Sr(a) costuma comer frutas? e “Em quantos dias da semana, o(a) Sr(a) costuma comer pelo menos um tipo de verdura ou legume?”. Assim, foi possível analisar a associação do consumo de carnes vermelhas e de frango com o consumo de alimentos in natura (frutas e hortaliças).

### Fonte de dados e mensuração

A coleta de dados ocorreu entre agosto de 2019 e março de 2020, em computadores de mão programados para evitar erros de digitação e questões sem resposta. O questionário era composto por 26 módulos, envolvendo informações dos domicílios, características dos moradores (educação, trabalho e renda), dados de cobertura e utilização de serviços de saúde, percepção do estado de saúde, acidentes, estilo de vida, doenças crônicas, atendimento pré-natal, saúde bucal, violências, doenças transmissíveis, atividades sexuais, condições de trabalho e antropometria. Para o desenvolvimento desta pesquisa, foram utilizadas informações dos módulos A (domicílio), C (características gerais dos moradores), D (características de educação dos moradores), E (características de trabalho dos moradores), F (rendimentos domiciliares), P (estilos de vida) e Y (atividade sexual). 

### Viés

Para garantia do controle e qualidade dos dados, toda a equipe de coleta foi treinada sistematicamente para a coleta de dados.

### Tamanho do estudo

Esse estudo é representativo do Brasil, macrorregiões, população urbana, rural e capitais. O seu processo amostral foi composto por amostragem aleatória simples, por conglomerados em três estágios: ^1^ unidades primárias de amostragem compostas por um ou mais setores censitários; ^2^ domicílios presentes em cada idades primárias de amostragem selecionada no primeiro estágio; ^3^ morador adulto (≥15 anos) selecionado em cada domicílio ^12^.

Para determinar o tamanho da amostra e garantir precisão das estimativas, foram considerados alguns indicadores da edição de 2013 da PNS. Esses indicadores incluíam dados sobre doenças crônicas não transmissíveis (como diabetes, hipertensão e depressão), violência, uso de serviços de saúde, posse de plano de saúde, tabagismo, prática de atividade física e consumo de álcool, entre outros. A pesquisa envolveu a seleção de 108.525 domicílios para participarem da entrevista. Entre esses, foram realizadas 90.846 entrevistas, resultando em uma amostra final. A taxa de resposta alcançada foi de 96,5%. 

Devido ao desenho amostral complexo, com probabilidades distintas de seleção da amostra, foram definidos fatores de expansão para domicílios e para os moradores selecionados, aqui chamados de pesos amostrais. O peso final é o produto do inverso das expressões de probabilidade de seleção de cada estágio do plano amostral, incluindo correção de não respostas e ajustes dos totais populacionais ^12^. 

### Métodos estatísticos

Foram realizadas análises descritivas e inferenciais. Para isso, foram calculadas frequências absolutas e relativas e seus respectivos intervalos de confiança de 95% (IC95%) para descrever as variáveis. 

Para testar as associações, foram utilizadas análises bivariadas, utilizando teste estatísticos de qui-quadrado de Pearson com ajuste Rao e Scott. Por fim, foram utilizados modelos lineares generalizados com distribuição logística multinomial e de Poisson, para testar o efeito individual e associado das variáveis explicativas nas variáveis dependentes. Razões de chances (odds ratio, OR) e seus respectivos IC95% foram calculadas a partir dos coeficientes estimados pelos modelos logísticos multinomiais.

Todas as análises foram realizadas com uso da a linguagem R, através da interface R Studio. Os dados foram ajustados de acordo com os respectivos pesos amostrais, utilizando-se o pacote Survey ^13^ e PNSIBGE ^14^.

## Resultados

A Tabela 1 descreve as características sociodemográficas e econômicas da população segundo tercis de consumo de carnes vermelhas. Aproximadamente 25 mil (29,3%) participantes relataram consumir carne 5-7 vezes na semana (terceiro tercil de consumo). Quando investigados os fatores sociodemográficos e econômicos, foi verificado que maior consumo semanal (5-7 vezes) foi mais prevalente entre os homens, quando comparados às mulheres (56,2% versus 43,8%, p-valor<0,001); negros, quando comparados aos não negros (52,4% vs. 47,6%, p-valor<0,001); residentes em áreas urbanas, comparados a residentes em áreas rurais (85,1% vs. 14,9%, p-valor<0,001); e maior escolaridade - superior (20,0%), médio (37,2%) e fundamental (27,7%), em comparação com aqueles no ensino primário ou menos (15,1%; p-valor<0,001). Em relação à renda, os indivíduos no primeiro tercil de renda apresentaram consumo menos frequente de carne, quando comparados àqueles do terceiro tercil (16,9 vs. 50,9%, p-valor<0,001). Não foram observadas diferenças quanto à orientação sexual.

**Tabela 1:** Frequência de consumo de carne vermelha em dias (%) e intervalo de confiança de 95% (IC95%) por fatores socioeconômicos na população. Brasil, 2019 (n=83.085)

Variável	<2 dias	2-4 dias	5-7 dias	p-valor
**Sexo**				<0,001
Masculino	39,3 (38,4; 40,2)	47,5 (46,6; 48,4)	56,5 (55,1; 57,2)	
Feminino	60,7 (59,8; 61,6)	52,5 (51,6; 53,3)	43,8 (42,8; 44,9)	
**Orientação sexual**				0,064
Heterossexual	95,3 (94,9; 95,8)	95,7 (95,2; 96,1)	94,9 (94,43; 95,48)	
Homo e bissexual	1,7 (1,4; 1,9)	1,7 (1,4; 1,9)	1,6 (1,4; 1,9)	
Outros	3,0 (2,7; 3,3)	2,7 (2,34; 3,00)	3,4 (2,9; 3,9)	
**Raça/cor da pele**				<0,001
Negros	57,4 (56,3; 58,5)	52,8 (51,7; 53,9)	52,4 (51,3; 53,5)	
Não negros	42,6 (41,5; 43,7)	47,2 (46,1; 48,3)	47,6 (46,5; 48,7)	
**Escolaridade**				<0,001
Ensino primário ou menor	18,2 (17,4; 19,1)	14,7 (14,0; 15,4)	15,1 (14,3; 15,9)	
Ensino fundamental	29,0 (28,0; 29,9)	25,6 (24,6; 26,6)	27,7 (26,6; 28,7)	
Ensino médio	35,2 (34,2; 36,2)	36,9 (35,9; 37,9)	37,2 (36,0; 38,3)	
Ensino superior	17,6 (16,6; 18,6)	22,8 (21,6; 23,9)	20,0 (18,8; 21,2)	
**Situação**				<0,001
Urbano	86,2 (85,6; 86,7)	87,2 (86,5; 87,7)	85,1 (84,4; 85,9)	
Rural	13,8 (13,2; 14,5)	12,9 (12,3; 13,5)	14,9 (14,2; 15,6)	
**Região**				<0,001
Norte	8,5 (8,1; 8,9)	7,0 (6,7; 7,4)	6,6 (6,2; 6,9)	
Nordeste	30,9 (29,9; 31,7)	25,2 (24,3; 26,1)	19,9 (18,9; 20,7)	
Sudeste	44,3 (43,2; 45,5)	44,5 (43,3; 45,7)	41,1 (39,9; 42,4)	
Sul	12,2 (11,6; 12,8)	16,4 (15,7; 17,1)	19,3 (18,4; 20,2)	
Centro-Oeste	4,2 (3,9; 4,4)	6,9 (6,5; 7,3)	13,2 (12,5; 13,9)	
**Renda**				<0,001
1^o^ tercil	26,1 (25,1; 26,9)	19,8 (19,0; 20,5)	16,9 (16,6; 17,6)	
2^o^ tercil	32,9 (31,9; 33,8)	31,4 (30,4; 32,4)	32,9 (31,2; 33,2)	
3^o^ tercil	41,1 (40,0; 42,2)	48,8 (47,6; 50,1)	50,9 (49,7; 52,7)	
**Idade**				<0,001
Adolescentes	3,8 (3,4; 4,2)	4,2 (3,8; 4,6)	4,6 (4,2; 5,1)	
Adultos	69,4 (68,5; 70,4)	73,6 (72,6; 74,5)	73,6 (72,6; 74,5)	
Idosos	26,8 (25,8; 27,7)	22,2 (21,3; 23,1)	21,8 (20,9; 22,7)	

Na Tabela 2, observa-se que o grupo com menor chance de consumo de carne igual a 5-7 dias/semana é o de mulheres [razão de chances (odds ratio, OR) 0,50, p-valor<0,001), os idosos (OR 0,48, p-valor<0,001) e os residentes do Nordeste do país (OR 0,72, p-valor<0,001). Por outro lado, a maior chance de um consumo de carne 5-7 dias/semana se deu entre os habitantes das regiões Sul (OR 1,63, p-valor<0,001) e Centro-Oeste (OR 3,62, p-valor<0,001), com maior renda (OR 1,65, p-valor<0,001), que cursaram o ensino médio (OR 1,14, p-valor<0,014) e que viviam em área rural (OR 1,37, p-valor<0,001) (Tabela 2).

**Tabela 2:** Razão de chances (odds ratio, OR) e intervalo de confiança de 95% (IC95%) de consumo de carne por variáveis sociodemográficas na população. Brasil, 2019 (n=83.085)

Variável	Frequência de consumo de carnes vermelhas			
	2-4 dias	p-valor	5-7 dias	p-valor
	OR (IC95%)		OR (IC95%)	
				
**Sexo**				<0,001
Masculino	1,00		1,00	
Feminino	0,70 (0,66; 0,74)	<0,001	0,50 (0,46; 0,53)	<0,001
**Idade (anos)**				
Adolescentes (0-19)	1,00		1,00	
Adultos (19-60)	0,79 (0,61; 1,02)	0,074	0,66 (0,52; 0,85)	0,001
Idosos (≥60)	0,63 (0,49; 0,82)	<0,001	0,48 (0,37; 0,63)	<0,001
**Raça/cor da pele**				
Negros	1,00		1,00	
Não negros	0,95 (0,89; 1,01)	<0,11	0,95 (0,89; 1,02)	<0,200
**Escolaridade**				
Ensino primário ou menor	1,00		1,00	
Ensino fundamental	1,03 (0,93; 1,13)	0,6	1,08 (0,97; 1,19)	0,2
Ensino médio	1,18 (1,07; 1,30)	<0,001	1,14 (1,03; 1,27)	<0,014
Ensino superior	1,36 (1,21; 1,52)	<0,001	1,11 (0,98; 1,25)	0,110
**Situação**				
Urbano	1,00		1,00	
Rural	1,10 (1,0; 1,19)	0,016	1,37 (1,26; 1,50)	<0,001
**Região**				
Sudeste	1,00		1,00	
Norte	0,87 (0,79; 0,95)	0,003	0,86 (0,77; 0,96)	0,007
Nordeste	0,88 (0,82; 0,96)	0,002	0,72 (0,66; 0,79)	<0,001
Sul	1,33 (1,22; 1,46)	<0,001	1,63 (1,48; 1,80)	<0,001
Centro-Oeste	1,67 (1,51; 1,86)	<0,001	3,62 (3,23; 4,04)	<0,001
**Renda**				
1^o^	1,00		1,00	
2^o^	1,20 (1,10; 1,30)	<0,001	1,40 (1,29; 1,52)	<0,001
3^o^	1,32 (1,20; 1,44)	<0,001	1,65 (1,50; 1,82)	<0,001

Após ajuste por sexo, idade, escolaridade, situação censitária, região do país e renda, o consumo de carne foi inversamente associado ao consumo de frutas (β -0,04) e diretamente associado ao consumo de hortaliças (β 0,04) (Tabela 3).

**Tabela 3:** Estimativa (β) e erro-padrão (EP) da frequência de consumo de frutas e hortaliças de acordo com a frequência de consumo de carnes vermelhas na população. Brasil, 2019 (n=83.085)

	Frutas		Hortaliças	
	β (EP)	β ajustado^a^ (EP)	β (EP)	β ajustado^a^ (EP)
**Frequência no consumo de carnes vermelhas (dias/semana)**				
0-1	1	1	1	1
2-4	0,01 (0,01)	0,01 (0,01)	0,05 (0,01)^b^	0,04 (0,01)^b^
5-7	-0,05 (0,01)^b^	-0,04 (0,01)^b^	0,05 (0,01)^b^	0,04 (0,01)^b^

Notas: ^a^Ajustado por sexo, idade, escolaridade, situação censitária, região do país e renda; bp-valor<0,001.

## Discussão

A análise dos dados revelou desigualdades de sexo, raça/cor da pele, região e renda no consumo de carne vermelha entre os brasileiros, e que o consumo de carne vermelha pode ter impactado negativamente o consumo de frutas. Mulheres, pessoas idosas e residentes no Norte e Nordeste do país apresentaram menos chance de consumo de carne vermelha. Enquanto pessoas com maior renda que residiam na região Centro-Oeste do país apresentaram maior chance de consumo de carne vermelha, quando comparados a suas contrapartes. 

As disparidades no consumo de carnes mostram que o maior impacto ambiental relacionado ao consumo de carnes está concentrado principalmente em grupos sociais com maiores níveis de privilégio social. Os resultados deste artigo reforçam a relação entre aspectos socioculturais e o consumo de carne, mostrando um maior consumo para homens brancos de renda elevada e residentes em regiões de maior desenvolvimento econômico. Apesar de não termos avaliado o impacto ambiental do consumo em questão, as carnes são os alimentos que mais contribuem para os impactos negativos do sistema alimentar no meio ambiente, de forma que o seu consumo elevado está diretamente relacionado com piores indicadores ambientais ^6^.

A associação positiva entre consumo de carne vermelha e hortaliças indicou que, quanto maior o consumo de carne vermelha, maior o consumo de hortaliças. Esse achado reforça a hipótese de que no Brasil, culturalmente, as carnes vermelhas são consumidas durante as refeições principais, almoço e jantar, e estão associadas a preparações culinárias ^15^. Por sua vez, a associação negativa entre o consumo de carne vermelha e frutas pode ser explicada pelo alto custo da carne para grupos de menor renda ^7^. Assim, as frutas podem estar sendo preteridas em detrimento da aquisição de carne ou de outros alimentos com capacidade de ofertar maior consumo energético e que possuem maior valor social no prato do brasileiro ^15^ ^,^ ^16^.

A recomendação para redução do consumo de carnes, entretanto, não pode ser universal, frente às profundas desigualdades sociais no Brasil. Dietas com menor frequência do consumo de carnes vermelhas por mulheres e pessoas vivendo no Norte e Nordeste podem estar relacionadas ao impacto das desigualdades sociais na alimentação e nutrição no Brasil. A insegurança alimentar e a redução do consumo de carne estão correlacionados ^17^. Em 2021/2022, 58,7% dos domicílios brasileiros estavam em insegurança alimentar. Desses, 19,3% eram lares comandados por mulheres que estavam em insegurança alimentar. Quase 8% a mais do que o observado em lares comandados por homens. Ainda, as maiores prevalências de insegurança alimentar foram mensuradas no Norte (45,2%) e no Nordeste (38,4%) ^18^.Dessa forma, as recomendações sobre consumo de carne devem considerar duas mensagens distintas para grupos sociais em polos opostos: 1) a garantia da segurança alimentar e nutricional para pessoas em vulnerabilidade social e que acessam na carne nutrientes essenciais; e 2) a redução do consumo de carne para grupos em segurança alimentar e nutricional, que vivem em um contexto de possibilidade de escolhas alimentares sustentáveis.

Desigualdades de consumo também foram observadas quanto à renda. Indivíduos no terceiro tercil de renda, quando comparados aos do primeiro tercil, apresentaram maior chance de frequência de consumo de carne vermelha de 5-7 vezes/semana. Esta tendência é observada em toda a América Latina, de forma que o aumento da renda impulsiona o consumo de carne em uma velocidade superior a países ricos ^19^. Apesar de novas evidências mostrarem que, em uma perspectiva global, a relação entre carne e renda tem um formato de U invertido, o ponto de inflexão é tão alto que se encontra acima do percentil 90 para a renda per capita ^20^. Em termos práticos, intervenções para a redução do consumo de carnes deveriam focar populações com maior renda.

Por outro lado, a relação entre idade e menor chance do consumo de carne não parece ter uma justificativa pautada em injustiça social. Pesquisas indicam que adultos mais velhos tendem a comer menos carne, devido a vários fatores. A percepção sensorial e as preferências alimentares mudam com a idade, afetando o consumo de carne. A dificuldade em comer produtos de carne, especialmente aqueles com texturas duras, é um problema comum para adultos mais velhos ^21^.

Desenvolver recomendações alimentares nacionais baseadas em sustentabilidade é um desafio para contextos de desigualdade social. A carne é um alimento que desafia simultaneamente dois princípios do Guia Alimentar para a População Brasileira: o consumo de alimentos in natura e a sustentabilidade alimentar. O consumo de carne vermelha está diretamente ligado a maiores riscos de excesso de gordura saturada e sódio, mas ajuda a prevenir a insuficiência de nutrientes essenciais, como proteínas, ferro, zinco e vitamina B12 ^22^. Por outro lado, para atender o princípio da sustentabilidade ambiental, o consumo de carne precisa ser limitado, pois é responsável pela maior parte das emissões de gases de efeito estufa e gasto de água provenientes dos sistemas alimentares ^5^. No entanto, uma restrição do consumo de carnes pode representar um aumento em 10% na desnutrição de grupos em vulnerabilidade ^10^. Comunicar este paradigma para a população brasileira parece um dos desafios urgentes para promoção da alimentação saudável e sustentável.

Contudo, esses achados devem ser analisados à luz de algumas limitações. A limitação principal deste estudo é o viés de informação presente na frequência de consumo autorrelatada, uma característica da PNS. Isso não limita a força e a interpretação dos resultados do artigo, que se trata de um dos primeiros estudos de amostra nacional sobre as desigualdades do consumo de carne vermelha e fatores associados.

Apesar das limitações metodológicas, o estudo destaca a importância de utilizar grandes bases de dados nacionais, como a PNS, para realizar análises abrangentes e representativas. A utilização de uma amostra nacional robusta e a aplicação de métodos estatísticos avançados fortalecem a validade dos resultados e a sua relevância para a formulação de políticas de saúde. 

Conclui-se que o presente estudo oferece insights preciosos sobre a relação entre fatores sociodemográficos e os padrões alimentares, e indica possibilidades futuras de análise. Seus resultados possibilitam implicações mais amplas para a saúde pública do país, ao serem avaliados fatores demográficos e socioeconômicos, como também indicam os desafios para adesão ao Guia Alimentar, sua regra de ouro e seus princípios, em um cenário de desigualdades sociais e econômicas.

Desse modo, essas informações são imprescindíveis, em paralelo àquelas sobre gastos com carnes segundo desigualdades, para o desenvolvimento de políticas públicas e intervenções de saúde direcionadas, que visem reduzir desigualdades e promover uma alimentação mais saudável.

Além disso, a relação identificada entre o consumo de carne vermelha e o consumo de alimentos in natura, como as frutas e hortaliças, fornece bases para se promover uma alimentação adequada, saudável e sustentável. Ao compreenderem os padrões de consumo alimentar e os fatores que os influenciam, os formuladores de políticas podem desenvolver estratégias que incentivem a substituição de carne vermelha por opções mais saudáveis e sustentáveis, contribuindo para a melhoria da saúde pública e a sustentabilidade ambiental. Desse modo, esta perspectiva é especialmente relevante no contexto atual, em que as doenças crônicas não transmissíveis e a degradação ambiental são preocupações crescentes.

## Data Availability

Não se aplica
